# Brain Structural Covariance Networks in Behavioral Variant of Frontotemporal Dementia

**DOI:** 10.3390/brainsci11020192

**Published:** 2021-02-04

**Authors:** Salvatore Nigro, Benedetta Tafuri, Daniele Urso, Roberto De Blasi, Maria Elisa Frisullo, Maria Rosaria Barulli, Rosa Capozzo, Alessia Cedola, Giuseppe Gigli, Giancarlo Logroscino

**Affiliations:** 1 Institute of Nanotechnology (NANOTEC), National Research Council, 73100 Lecce, Italy; salvatore.nigro@nanotec.cnr.it (S.N.); alessia.cedola@cnr.it (A.C.); giuseppe.gigli@cnr.it (G.G.); 2 Center for Neurodegenerative Diseases and the Aging Brain, Department of Clinical Research in Neurology, University of Bari ‘Aldo Moro, “Pia Fondazione Cardinale G. Panico”, 73039 Tricase, Italy; benedetta.tafuri@gmail.com (B.T.); danieleurso010@gmail.com (D.U.); robertodeblasi@hotmail.com (R.D.B.); m.frisullo@piafondazionepanico.it (M.E.F.); orietta.barulli@gmail.com (M.R.B.); rcapozzo@piafondazionepanico.it (R.C.); 3 Department of Neurosciences, King’s College London, Institute of Psychiatry, Psychology and Neuroscience, De Crespigny Park, London SE5 8AF, UK; 4 Department of Radiology, “Pia Fondazione Cardinale G. Panico”, 73039 Tricase, Italy; 5 Department of Mathematics and Physics “Ennio De Giorgi”, University of Salento, Campus Ecotekne, 73100 Lecce, Italy; 6 Department of Basic Medicine, Neuroscience, and Sense Organs, University of Bari ‘Aldo Moro’, 70124 Bari, Italy

**Keywords:** behavioral variant frontotemporal dementia, structural covariance network, graph analysis, MRI

## Abstract

Recent research on behavioral variant frontotemporal dementia (bvFTD) has shown that personality changes and executive dysfunctions are accompanied by a disease-specific anatomical pattern of cortical and subcortical atrophy. We investigated the structural topological network changes in patients with bvFTD in comparison to healthy controls. In particular, 25 bvFTD patients and 20 healthy controls underwent structural 3T MRI. Next, bilaterally averaged values of 34 cortical surface areas, 34 cortical thickness values, and six subcortical volumes were used to capture single-subject anatomical connectivity and investigate network organization using a graph theory approach. Relative to controls, bvFTD patients showed altered small-world properties and decreased global efficiency, suggesting a reduced ability to combine specialized information from distributed brain regions. At a local level, patients with bvFTD displayed lower values of local efficiency in the cortical thickness of the caudal and rostral middle frontal gyrus, rostral anterior cingulate, and precuneus, cuneus, and transverse temporal gyrus. A significant correlation was also found between the efficiency of caudal anterior cingulate thickness and Mini-Mental State Examination (MMSE) scores in bvFTD patients. Taken together, these findings confirm the selective disruption in structural brain networks of bvFTD patients, providing new insights on the association between cognitive decline and graph properties.

## 1. Introduction

Behavioral variant frontotemporal dementia (bvFTD) is the most common frontotemporal lobar degeneration (FTLD), accounting for more than 50% of patients with autopsy-confirmed FTLD [1]. Characterized by a progressive impairment in social function and personality [2], patients with bvFTD often show a focused atrophy in several cortical and subcortical regions, such as the anterior cingulate, insula, prefrontal cortex, anterior temporal regions, striatum, and thalamus [3,4,5]. Despite the typical clinical features and anatomical changes, bvFTD remains difficult to diagnose, and may be confused with other neurological or psychiatric disorders [6]. Thus, the development of new biomarkers to enhance the diagnostic validity of bvFTD is crucial, particularly in the early stages of the clinical work-up and for the selection of participants to pharmacological and clinical trials.

In the last decade, neuroimaging studies have supported the idea that brain abnormalities observed in several neurodegenerative diseases not only involve changes in discrete brain regions, but are also characterized in terms of an altered organization in several functionally and anatomically interconnected regions [7,8,9]. Graph theoretical methods applied to resting state fMRI (rs-fMRI) and diffusion tensor imaging (DTI) data have also allowed the modeling of the brain as a complex network, revealing important features of global and local brain organization [10,11,12,13]. In this context, previous studies have revealed that brain networks in healthy controls show a small-world topology, supporting both specialized and integrated information processing [10,14]. Although small-world properties have also been observed in patients with several brain disorders, alterations in these network measures have been reported in comparison with healthy controls [9,14,15,16,17,18,19,20,21].

In recent years, fMRI- and DTI-based brain connectivity measures have been complemented by a novel class of measurements using the inter-individual or intra-individual covariation in brain morphology (e.g., volume, thickness, or surface area) to characterize structural connectivity between regions and define large-scale brain networks (i.e., structural covariance networks) [22,23,24]. A crucial assumption underlying this procedure is that the morphological properties of interconnected brain regions would covary, since they share common developmental and maturational influences [22]. Structural covariance networks have demonstrated connectivity patterns similar to those detected in functional and diffusion-based anatomical networks [25,26]. At the same time, their construction is less sensitive to noise in comparison to those of functional and DTI-based networks [27,28], and requires relatively lower computational loads [29]. Although several studies have used brain structural covariance and graph analysis in neurodegenerative diseases [14,29,30,31,32,33], the investigation of changes in global and regional network characteristics within patients with bvFTD is still limited. To our knowledge, only one study has combined graph analysis and gray matter intensities to examine intra-individual structural covariation of the brain in bvFTD patients [34]. In that study, patients with bvFTD showed lower values of small-world properties compared to healthy controls. Lower MMSE scores were also associated with lower integration values in the right hippocampus.

In the current study, we used a new approach to human brain network mapping that leverages the increasing ability to obtain multiple morphological features from cortical and subcortical brain regions [33]. In particular, we used cortical thickness values, cortical surface areas, and subcortical volumes to capture single-subject anatomical connectivity and investigate network topology, applying a graph theory approach. We hypothesized that the structural covariance networks of patients with bvFTD should have altered global and local network properties compared to healthy controls. Considering the previously reported functional alterations and pathological damages in bvFTD, we expected changes in specific brain regions belonging to frontal and temporal circuits, which have been associated with bvFTD. To test this hypothesis, we employed the following local graph metrics: (i) a centrality measure (i.e., degree centrality), (ii) segregation measures (i.e., clustering coefficient), and (iii) integration measures (i.e., characteristic path length and local efficiency).

## 2. Materials and Methods

### 2.1. Participants

We included 25 bvFTD patients (14 males/11 females; 66.92 ± 7.69 years) and 20 control subjects (seven males/13 females; 63.60 ± 5.90 years). All participants were referred to the Neurodegerative Diseases Unit, Department of Clinical Research in Neurology of the University of Bari “Aldo Moro” at “Pia Fondazione Cardinale G. Panico”. According to Rascovsky’s criteria [2], patient diagnoses were based on a comprehensive evaluation, including clinical history, neurological examination, and neuropsychological testing. Eligibility criteria included no history of other neurological or psychiatric illnesses, no clinical or neuroimaging evidence of focal lesions, and no inflammatory, infectious, or vascular diseases. The control group was selected according to ADNI-3 criteria (ADNI Protocol v1.0: 24 May 2016, http://adni.loni.usc.edu/wp-content/themes/freshnews-dev-v2/documents/clinical/ADNI3_Protocol.pdf). None of the controls had a history of neurologic or psychiatric illness. The Mini-Mental State Examination (MMSE) and Frontal Assessment Battery (FAB) were administered to all participants as the screening assessment [35,36]. All participants gave written informed consent. The study was conducted according to the guidelines of the Declaration of Helsinki, and approved by the Institutional Review Board (or Ethics Committee) of ASL Lecce (verbale n. 6, 25 July 2017).

### 2.2. MRI Acquisition and Processing

Neuroimaging data were acquired on a 3T scanner (Philips Ingenia 3T). Set acquisition was in the sagittal plane using a Fast-Field Echo (FFE) T1-weighted sequence with the following parameters: repetition time = 8.2 ms, echo time = 3.8 ms, field of view = 256 × 256 mm^2^, 200 slices, flip angle = 8°, and isotropic 1 mm^3^ voxels.

T1-weighted images were inspected visually to check for motion-related artifacts and gross neuroanatomical alterations by a consultant neuroradiologist. Next, images were analyzed using FreeSurfer (version 6.0) (http://www.nmr.mgh.harvard.edu/martinos) to extract morphological features for cortical and subcortical brain regions [37,38,39]. Briefly, the cortical surface for each participant was reconstructed from T1-weighted images by the following steps: skull stripping, segmentation of cortical gray and white matter, separation of the two hemispheres and subcortical structures, and, finally, construction of smooth representation of the gray/white interface and the pial surface [37,39,40,41]. Next, all images were checked for reconstruction cortical surface errors, and surface inaccuracies were corrected with FreeSurfer’s editing tools. Then, the surface area was calculated using triangular tessellation of the gray/white matter interface and white matter/cerebrospinal fluid boundary (pial surface) [42]. Cortical thickness was also calculated based on the distance between closest points, between gray and white matter surfaces [39]. Finally, we used the FreeSurfer parcellation scheme based on the Desikan–Killiany Atlas to extract the cortical thickness and surface area of 68 cortical regions from both hemispheres [43]. Subcortical volumetric analyses were also performed using an automated approach that estimates the probability of structure classification based on prior templates in which those structures were manually identified [44]. We considered 12 subcortical areas, including the putamen, caudate, thalamus, pallidum, hippocampus, and amygdala, for each hemisphere. A list of cortical and subcortical regions is given in Supplementary Materials (Table S1).

### 2.3. Network Construction

Cortical thickness, surface area, and volumetric values were bilaterally averaged and corrected for age, sex, and individual brain size [33]. The resulting residuals were then z-score transformed using the mean and standard deviation values of each brain region calculated from healthy controls. Finally, a measure of joint variation between the 74 morphometric features (34 cortical surface area values, 34 cortical thickness values, and six subcortical volume values) represented the edge weights of the network, and was calculated using the following formula [33,45]:

1/exp{[(z-score value of ith region of interest) − (z-score value of jth region of interest)]^2^} (1)

### 2.4. Graph Theory Analysis

Estimation of the global and local network characteristics was performed by using the Graph Theoretical Network Analysis (GRETNA) (www.nitrc.org/projects/gretna/) packages [46]. Small world measures and global efficiency (Eglob) were used to characterize the global topological organization of the covariance structural networks in both controls and patients with bvFTD. In particular, to examine the small-world properties of a network, the normalized clustering coefficient γ = (Cp^real^/Cp^random^) and the normalized characteristic path length λ = (Lp^real^/Lp^random^) were first computed. Then, the small-world index was calculated as the ratio of the normalized clustering coefficient and the normalized path length (σ = γ/λ). Of note, Cp^real^ and Lp^real^ are the clustering and the characteristic path length of the real network, respectively, and Cp^random^ and Lp^random^ represent, respectively, the mean clustering coefficient and shortest path length of 1000 matched random networks that preserve the same numbers of nodes, edges, and degree distribution as the real network. A real network can be considered as a small-world network if it fulfills the following criteria: small-world index σ = λ/ γ > 1.1 [47,48]. Compared to a random network, a small-world network is thus characterized by a higher clustering coefficient. By contrast, it exhibits a short characteristic path length comparable to that of a random network.

Regional network properties were assessed using degree centrality, the clustering coefficient, local path length, and local efficiency [11,49,50,51]. Degree centrality is a local graph measure that is able to quantify the relative importance of a node within a network [51]. The clustering coefficient represents the ability of a node to communicate with other nodes with which it shares a direct connection (segregation ability) [49]. Nodal efficiency and characteristic path length, on the other hand, quantify the ability of information propagation between a node and the remaining nodes in the network (integration ability). Local efficiency is calculated as the global efficiency of the subgraph formed by the node’s neighbors. A node with high nodal efficiency or low path length indicates high capability of information transmission with other nodes. Detailed formulas and explanations of these global and local metrics can be found in previous methodological reviews [13,21,50].

As graph measures are non-trivially dependent on the density of the underlying graph [52], intra-individual structural covariance networks were thresholded in a network density range of d = 0.10–0.40, with an interval of 0.01. Connectivity thresholding is commonly used to remove noisy or spurious links, preserving the strongest structural edges. The range of density was chosen to allow small-world network properties to be properly estimated and the number of spurious edges in each network to be minimized [53,54]. Then, the network parameters were computed for each network at each density. Finally, GRETNA was used to calculate the area under the curve (AUC, i.e., the integral over the density range) for each network measure to provide a scalar that does not depend on specific threshold selection [55,56]. Of note, graph measures were calculated based on weighted structural networks. In this way, we could characterize the relative importance of each link between network nodes. The BrainNet Viewer (http://www.nitrc.org/projects/bnv/) was used to visualize the regional brain network changes between patients and healthy controls [57].

### 2.5. Statistical Analyses

The Shapiro–Wilk test was performed in either demographic, neuroimaging, and neuropsychological variables (i.e., age, total intracranial volume, cognitive performance), or graph measures to verify the normality of data distribution. Next, variables with a normal distribution were compared between controls and bvFTD patients using pairwise *t*-tests. Non-normally distributed variables were compared between groups using Wilcoxon–Mann–Whitney test. The chi-square test was used to test for differences in the sex distribution between groups. The critical statistical threshold was set to *p* < 0.05. A false discovery rate (FDR) correction procedure was employed to correct for multiple comparisons in the global and local network analyses [58]. The relationships between network metrics and clinical data (disease duration and cognitive performances) of patients with bvFTD were tested using the Pearson correlation (*p*-value < 0.05). The correlations were considered statistically significant if the relative *p*-values were less than 0.05 after FDR correction.

## 3. Results

### 3.1. Demographic and Clinical Characteristics

No differences were found in age, sex, years of education, or intracranial volume between the bvFTD patients and healthy controls (*p* > 0.05). Concerning clinical data, patients with bvFTD had significantly lower MMSE and FAB scores compared with healthy control participants (*p*-value < 0.001) (Table 1).

### 3.2. Global Network Characteristics

The structural covariance network of controls and bvFTD patients demonstrated small-world network architecture over the preselected density range (1.37 < σ_HC_ < 2.92; 1.25 < σ_bvFTD_ < 2.76). However, the small-world index was smaller in bvFTD patients than in controls. Moreover, the normalized characteristic path length values in patients were greater than those of controls (Table 2, *p* < 0.05, FDR corrected). Compared with the healthy control participants, the bvFTD group also exhibited significantly less global efficiency (Table 2, *p*-value < 0.001, FDR corrected). No significant difference was found in the normalized clustering coefficient values between bvFTD patients and healthy controls.

### 3.3. Regional Network Characteristics

**Figure 1:**
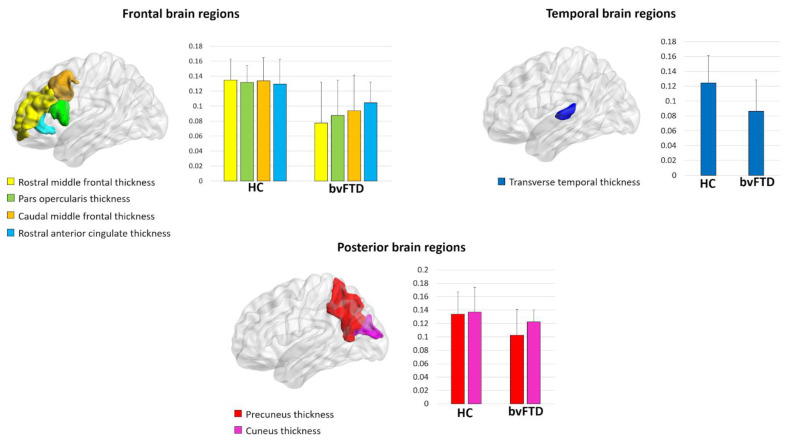
Regions showing decreased local efficiency in behavioral variant frontotemporal dementia (bvFTD) patients compared to healthy controls (HC).

At a local level, bvFTD patients displayed a reduced local efficiency in the cortical thickness of the rostral and caudal middle frontal gyrus, pars opercularis, precuneus, cuneus, transverse temporal gyrus, and rostral anterior cingulate (*p*-value < 0.05, FDR corrected) (Figure 1, Table 3). Moreover, we observed a reduced clustering in the cortical thickness of the inferior temporal gyrus in bvFTD patients compared with controls (*p*-value < 0.001, FDR corrected) (Table 3). No significant differences were found in the local properties of cortical surface areas and subcortical volumes between bvFTD patients and controls.

### 3.4. Correlation between Connectivity Metrics and Clinical Data

**Figure 2:**
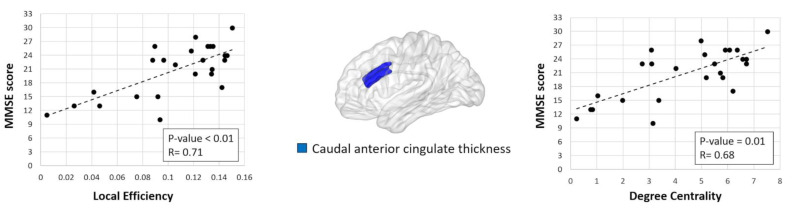
Significant correlation between degree centrality/nodal efficiency in the cortical thickness of the caudal anterior cingulate and Mini-Mental State Examination (MMSE) scores in behavioral variant frontotemporal dementia (bvFTD) patients.

Significant correlations were found in bvFTD patients’ MMSE scores with the local efficiency and nodal degree in the cortical thickness of the caudal anterior cingulate (Figure 2, *p*-value = 0.01, FDR corrected).

## 4. Discussion

In the present study, we applied graph analysis to investigate the topological organization of structural brain networks in patients with bvFTD. We found altered graph metrics both at a global and local level. More specifically, when compared to healthy controls, bvFTD patients showed altered small-world properties (i.e., increased normalized path length) and decreased global efficiency. At the local level, patients with bvFTD displayed lower values of local efficiency in cortical thickness of the caudal and rostral middle frontal gyrus, rostral anterior cingulate, and precuneus, cuneus, and transverse temporal gyrus. Relative to controls, patients with bvFTD also displayed reduced values of clustering coefficients in the thickness of the inferior temporal gyrus. Finally, a significant correlation was found between the efficiency of caudal anterior cingulate thickness and the MMSE scores in bvFTD patients.

Our findings provide new insights into our understanding of structural changes in the organization of bvFTD brain networks. In particular, the reduced small-world index (σ) observed in bvFTD patients suggests that the covariance networks of bvFTD patients tend to have a more randomized configuration compared to the control group [53]. Moreover, the disruption of both normalized path length and global efficiency is indicative of an impaired functional integration of bvFTD networks, indicating a reduced ability to combine specialized information from distributed brain regions [10,49]. In the past years, several studies have investigated small-world property alterations in healthy individuals [58,59,60], as well as in neurological and psychiatric disorders [17,19,32,61,62,63,64,65]. Neuroimaging studies have demonstrated that the cognitive and memory declines in Alzheimer’s disease patients are often associated with the disruption of the small-world structure [17,27,66,67]. Evidence from graph theoretical studies have also observed reduced functional and structural integrity in bvFTD brain networks when compared to healthy controls [16,17,34,68,69]. In line with these findings, the bvFTD-related global property alterations observed in the present study are thus suggestive of an impaired functional integration, which might contribute to impairments in the cognitive function of patients with bvFTD. This idea is further supported by local property changes that we found in the frontotemporal regions of bvFTD networks. Compared to controls, patients with bvFTD showed reduced local efficiency and clustering coefficients in the cortical thickness of the middle frontal gyrus, pars-opercularis, anterior cingulate, and temporal cortices. All of these regions represent the most prominent sites of bvFTD-related focal atrophy [4,5,70]. Moreover, they play a crucial role in executive control, working memory, and emotion processing that are often disrupted in bvFTD [71,72]. Decreased values in the local properties (i.e., nodal centrality, nodal strength) of frontotemporal regions were previously reported in functional and structural networks of patients with bvFTD in comparison to controls [16,68,69]. In the present study, the reduced ability in integration found in key regions of the frontotemporal network further confirm a strong involvement of this network in bvFTD pathophysiology. Furthermore, the local efficiency and centrality degree values of the cortical thickness in the caudal anterior cingulate were found to significantly and positively correlate with the MMSE score, indicating that the anterior cingulate might play a key role in driving cognitive deficits in bvFTD patients. Interestingly, we also found a reduced local efficiency in the cortical thickness of the precuneus and cuneus. Although gray matter alterations in these brain regions are not frequent in bvFTD patients, recent fMRI studies have reported functional connectivity alterations in posterior cortical areas of patients with FTD when compared to healthy controls, possibly reflecting reduced afferent input from limbic regions [73,74].

The current study has some limitations that need to be addressed. We considered a cohort of bvFTD patients without a histopathological confirmation. However, clinical examination was performed according to the most recent diagnostic criteria for FTD. Second, we examined a relatively small number of patients. Hence, a larger sample size is required to replicate our results. Third, in the calculation of intra-individual structural covariance networks, we used the bilaterally averaged values of cortical and subcortical morphological features. Therefore, we were not able to explore the homologous connectivity between the brain regions. However, bvFTD is traditionally associated with largely symmetrical atrophy of the frontal and temporal lobes [5,68]. Fourth, network measures are generally related to each other. Thus, it becomes difficult to say which of these measures is driving the others. The obtained results should therefore be interpreted with caution. Fifth, longitudinal studies are required to assess whether topological changes in the structural covariance network of bvFTD patients are predictive of clinical–pathological progression. Finally, it remains to be determined whether the local property changes that we found in the frontotemporal regions of bvFTD networks may represent a useful marker in distinguishing between FTD subtypes.

## 5. Conclusions

Our study provides new evidence for the usefulness of combining several morphometric measures to capture single-subject anatomical connectivity and then investigating bvFTD-related network organization using a graph theory approach. Compared to controls, patients with bvFTD showed altered graph metrics both at a global and local level. In particular, bvFTD patients were characterized by lower local efficiency values in the cortical thickness of several frontotemporal regions. These network alterations might contribute to cognitive impairments often observed in patients with bvFTD, as suggested by correlations between graph measures and MMSE scores.

## Figures and Tables

**Table 1:** Demographic, clinical, and neuroimaging data of sample.

	HC (n = 20)	bvFTD (n = 25)	*p*-Value	T/z
**Demographic and clinical data**				
**Age at exam (years)**	63.60 ± 5.90	66.92 ± 7.69	0.08	−1.74
**Sex (males/females)**	7/13	14/11	0.16	1.97
**Education (years)**	10.50 ± 4.88	8.32 ± 5.18	0.08	1.72
**MMSE**	27.90 ± 1.68	20.80 ± 5.57	<0.001	4.78
**FAB (z-score)**	−0.55 ± 0.95	−4.81 ± 3.60	<0.001	4.71
**Duration (years)**	-	2.86 ± 1.78	-	-
**Neuroimaging data**				
**Intracanial Volume (ml)**	1406.2 ± 155.71	1431.8 ± 163.69	0.81	−0.23

MMSE, Mini-Mental State Examination; FAB, Frontal Assessment Battery; HC, healthy controls; bvFTD, behavioral variant frontotemporal dementia patients.

**Table 2:** Main effect of the group in the global network metrics.

	HC (n = 20)	bvFTD (n = 25)	*p*-Value	T/z
σ	0.59 ± 0.04	0.55 ± 0.06	0.022	2.29
λ	0.42 ± 0.02	0.44 ± 0.02	0.008	−2.66
γ	0.86 ± 0.07	0.82 ± 0.09	0.09	1.70
Eglob	0.13 ± 0.01	0.12 ± 0.01	<0.001	4.42

All graph measure values are expressed as the area under the curve (AUC) across the density range; σ, small-world index; λ, normalized characteristic path length; γ, normalized clustering coefficient; Eglob, global efficiency; HC, healthy controls; bvFTD, behavioral variant frontotemporal dementia patients.

**Table 3:** Main effect of the group in the local efficiency and clustering coefficient.

Local Efficiency.				
Node	HC (n = 20)	bvFTD (n = 25)	*p*-Value FDR-Corrected	T/z
**Rostral middle frontal gyrus thickness**	0.14 ± 0.03	0.08 ± 0.05	0.02	3.69
Pars opercularis thickness	0.13 ± 0.02	0.09 ± 0.05	0.03	3.21
**Caudal middle frontal gyrus thickness**	0.13 ± 0.03	0.09 ± 0.05	0.03	3.16
Precuneus thickness	0.13 ± 0.03	0.10 ± 0.04	0.03	3.12
Cuneus thickness	0.14 ± 0.04	0.12 ± 0.02	0.04	3.00
Transverse temporal thickness	0.12 ± 0.04	0.09 ± 0.04	0.04	2.95
Rostral anterior cingulate thickness	0.13 ± 0.03	0.10 ± 0.03	0.05	2.84
**Clustering Coefficient**				
**Node**	**HC**	**bvFTD**	***p*** **-Value**	**T/z**
Inferior temporal gyrus thickness	0.23 ± 0.01	0.20 ± 0.02	<0.001	4.60

All graph measure values are expressed as the area under the curve (AUC) across the density range. HC, healthy controls; bvFTD, behavioral variant frontotemporal dementia patients.

## Data Availability

The data presented in this study are available on request from the corresponding author. The data are not publicly available due to privacy restrictions.

## Supplementary Materials

The following are available online at https://www.mdpi.com/2076-3425/11/2/192/s1, Table S1: List of cortical and subcortical brain regions used to construct structural covariance networks.
