# Supplementary material for: Brain Structural Covariance Networks in Behavioral Variant of Frontotemporal Dementia
Source: Brain Sci. 2021 Feb 4;11(2):192. doi: 10.3390/brainsci11020192 (PMC7915789; doi:10.3390/brainsci11020192)
Supplement: Supplementary file 1 [file brainsci-11-00192-s001.zip › brainsci-1058190-supplementary.pdf]

Table S1. List of cortical and subcortical brain regions used to construct structural covariance networks.

Cortical regions			
**Index**	**Name**	**Hemisphere**	**Lobe**
1	Bank of the superior temporal sulcus	L	Temporal
2	Caudal anteriocingulate	L	Cingulate
3	Caudal middle frontagyrus	L	Frontal
4	Cuneus	L	Occipital
5	Entorhinal	L	Temporal
6	Fusiform	L	Temporal
7	Inferior parietal lobule	L	Parietal
8	Inferior temporal gyrus	L	Temporal
9	Isthmus cingulate cortex	L	Cingulate
10	Lateral occipital gyrus	L	Occipital
11	Lateral orbitofrontal	L	Frontal
12	Lingual	L	Occipital
13	Medial orbitofrontal	L	Frontal
14	Middle temporal gyrus	L	Temporal
15	Parahippocampal	L	Temporal
16	Paracentral	L	Frontal
17	Pars opercularis	L	Frontal
18	Pars orbitalis	L	Frontal
19	Pars triangularis	L	Frontal
20	Pericalcarine	L	Occipital
21	Postcentral	L	Parietal
22	Posterior cingulate cortex	L	Cingulate
23	Precentral	L	Frontal
24	Precuneus	L	Parietal
25	Rostral anterior cingulate cortex	L	Cingulate
26	Rostral middle frontal gyrus	L	Frontal
27	Superior frontal gyrus	L	Frontal
28	Superior parietal lobule	L	Parietal
29	Superior temporal gyrus	L	Temporal
30	Supramarginal gyrus	L	Parietal
31	Frontal pole	L	Frontal
32	Temporal pole	L	Temporal
33	Transverse temporal	L	Temporal
34	Insula	L	Insula
35	Bank of the superior temporal sulcus	R	Temporal
36	Caudal anteriocingulate	R	Cingulate
37	Caudal middle frontagyrus	R	Frontal
38	Cuneus	R	Occipital
39	Entorhinal	R	Temporal
40	Fusiform	R	Temporal
41	Inferior parietal lobule	R	Parietal
42	Inferior temporal gyrus	R	Temporal
43	Isthmus cingulate cortex	R	Cingulate

44	Lateral occipital gyrus	R	Occipital
45	Lateral orbitofrontal	R	Frontal
46	Lingual	R	Occipital
47	Medial orbitofrontal	R	Frontal
48	Middle temporal gyrus	R	Temporal
49	Parahippocampal	R	Temporal
50	Paracentral	R	Frontal
51	Pars opercularis	R	Frontal
52	Pars orbitalis	R	Frontal
53	Pars triangularis	R	Frontal
54	Pericalcarine	R	Occipital
55	Postcentral	R	Parietal
56	Posterior cingulate cortex	R	Cingulate
57	Precentral	R	Frontal
58	Precuneus	R	Parietal
59	Rostral anterior cingulate cortex	R	Cingulate
60	Rostral middle frontal gyrus	R	Frontal
61	Superior frontal gyrus	R	Frontal
62	Superior parietal lobule	R	Parietal
63	Superior temporal gyrus	R	Temporal
64	Supramarginal gyrus	R	Parietal
65	Frontal pole	R	Frontal
66	Temporal pole	R	Temporal
67	Transverse temporal	R	Temporal
68	Insula	R	Insula
**Subcortical regions**			
1	Thalamus	L	Subcortical
2	Hippocampus	L	Subcortical
3	Amygdala	L	Subcortical
4	Putamen	L	Subcortical
5	Pallidum	L	Subcortical
6	Caudate	L	Subcortical
7	Thalamus	R	Subcortical
8	Hippocampus	R	Subcortical
9	Amygdala	R	Subcortical
10	Putamen	R	Subcortical
11	Pallidum	R	Subcortical
12	Caudate	R	Subcortical
